# Over-expression of chrysanthemum *CmDREB6* enhanced tolerance of chrysanthemum to heat stress

**DOI:** 10.1186/s12870-018-1400-8

**Published:** 2018-09-04

**Authors:** Xinping Du, Wenyan Li, Liping Sheng, Ye Deng, Yinjie Wang, Wanwan Zhang, Kaili Yu, Jiafu Jiang, Weimin Fang, Zhiyong Guan, Fadi Chen, Sumei Chen

**Affiliations:** College of Horticulture, Nanjing Agricultural University, Nanjing, 210095 China

**Keywords:** *CmDREB6*, Chrysanthemum, Abiotic stress, Heat tolerance

## Abstract

**Background:**

Chrysanthemum is among the top ten traditional flowers in China, and one of the four major cut flowers in the world, but the growth of chrysanthemum is severely restricted by high temperatures which retard growth and cause defects in flowers. DREB (dehydration-responsive element-binding) transcription factors play important roles in the response to abiotic and biotic stresses. However, whether the DREB A-6 subgroup is involved in heat tolerance has not been reported conclusively.

**Result:**

In the present study, *CmDREB6* was cloned from chrysanthemum (*Chrysanthemum morifolium*) ‘Jinba’. CmDREB6, containing a typical AP2/ERF domain, was classed into the DREB A-6 subgroup and shared highest homology with *Cichorium intybus* L. *CiDREB6* (73%). *CmDREB6* was expressed at its highest levels in the leaf. The CmDREB6 protein localized to the nucleus. Based on the yeast one hybrid assay, *CmDREB6* showed transcription activation activity in yeast, and the transcriptional activation domain was located in the 3 ‘end ranging from 230 to 289 amino acids residues. *CmDREB6* overexpression enhanced the tolerance of chrysanthemum to heat. The survival rate of two transgenic lines was as high as 85%, 50%, respectively, in contrast to 3.8% of wild-type (WT). Over-expression of *CmDREB6* promoted the expression of *CmHsfA4*, *CmHSP90,* and the active oxygen scavenging genes *CmSOD* and *CmCAT*.

**Conclusion:**

In this study, DREB A-6 subgroup gene *CmDREB6* was cloned from chrysanthemum ‘Jinba’. Overexpression of *CmDREB6* enhanced heat tolerance of chrysanthemum by regulating genes involved in the heat shock response and ROS homeogenesis.

**Electronic supplementary material:**

The online version of this article (10.1186/s12870-018-1400-8) contains supplementary material, which is available to authorized users.

## Background

Plants face various abiotic stresses, among which heat stress has become one of the main factors affecting crop growth, quality and yield. High temperatures hamper plant photosynthesis, causing cell membrane damage, cell aging and death [1]. Heat stress can also affect enzymatic activities, which in turn have a negative impact on plant growth and metabolism.

Plants and other organisms have both an inherent ability to survive exposure to temperatures above the optimal for growth (basal thermotolerance) and an ability to acquire tolerance to otherwise lethal heat stress (acquired thermotolerance), where acquired tolerance depends on the activation of a number of transcription factors [2]. DREB transcription factors belong to a subfamily of the AP2/EREBP transcription factors family, whose conserved AP2/EREBP (ethylene-responsive element binding proteins) domain plays a key role in the binding of DREB transcription factors to cis-acting elements of DREB. The DREB group is divided into 6 subgroups (A-1 to A-6), and the A-1 and A-2 subgroups contain DREB1s and DREB2s genes, respectively. In Arabidopsis, these two types of genes are involved in low temperature, drought and high salt stress responses [3]. Overexpression of *AtDREB1A* was found to enhance tolerance of chrysanthemum to heat [4]. However, whether the DREB A-6 subgroup is involved in heat tolerance has not been reported conclusively.

Generally, when the temperature is higher than an ambient temperature of 10–15 °C, plants will generate a heat shock response (HSR) quickly, within a few hours, in order to tolerate the otherwise lethal temperature [5]. The HSR is mediated at the transcriptional level by cis-acting sequences called heat shock elements (HSEs) that are present in multiple copies upstream of the heat shock protein (HSP) genes [6]. The heat shock factor (HSF) plays an important role in the regulation of the expression of HSP genes in plants. When the plants are subjected to heat stimulation, a HSF can specifically bind to a HSE, thus activating the HSP gene expression in vivo. The rapid accumulation of heat shock proteins in the plant helps the folding, stabilization and assembling of proteins, thus improving the tolerance of plants to high temperature [7].

Chrysanthemum is among the top ten traditional flowers in China, and one of the four major cut flowers in the world. However, the growth of chrysanthemum is severely restricted by high temperatures which retard growth and cause defects in flowers [8]. In this study, we cloned *CmDREB6*, a member of the DREB family of the A-6 subgroup, from chrysanthemum ‘Jinba’ and further analyzed its expression profiles and transactivation activities. We successfully generated *CmDREB6* overexpressing chrysanthemum lines and elucidated its regulatory roles in heat stress tolerance of chrysanthemum, thus shading a new light on the roles of the A-6 group DREB in abiotic stress tolerance.

## Results

### Cloning and phylogenetic analysis of *CmDREB6* from chrysanthemum

**Fig. 1:**
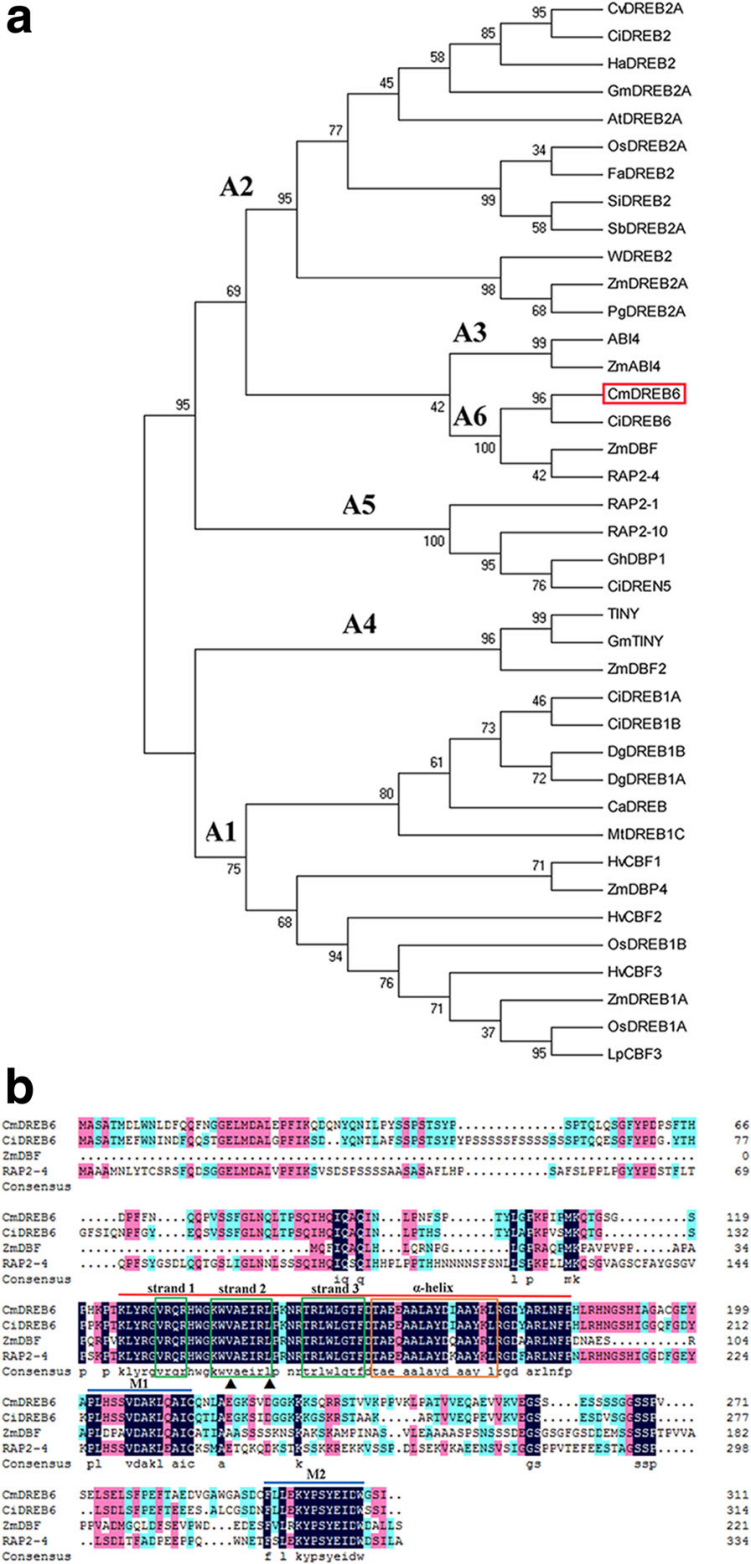
Phylogenetic analysis and alignment of the deduced amino acid sequences of the DREB peptide sequences. **a** The phylogenic relationship of CmDREB6 and 39 members from other species of CvDREB2A(ABR23508.1), CiDREB2(AHJ08574.1), HaDREB2(AAS82861.1), GmDREB2A(AFU35563.1), DREB2A(BAA33794.1), OsDREB2A(AAN02487.2), FaDREB2(AAR11157.1), SiDREB2(ADM73511.1), SbDREB2A(AEI69362.1), PeDREB2L(ABV03750.1), WDREB2(BAD97369.1), ZmDREB2A(BAE96012.1), PgDREB2A(AAV90624.1), ZmDBF(AAM80486.1), CiDREB6(AHJ08575.1), RAP2–4(NP_177931.1), ABI4(AAC39489.1), ZmABI4(AAM95247.1), RAP2–1(NP_564496.1), RAP2–10(NP_195408.1), GhDBP1(AAO43165.1), CiDREN5(AHJ08576.1), TINY(AAC29139.1), GmTINY(ACP40513.1), ZmDBF2(AAM80485.1), CiDREB1A(AHI59150.1), DgDREB1B(ABD90467.1), DgDREB1A(ABD90468.1), CiDREB1B(AHI59151.1), CaDREB(AAR88363.1), MtDREB1C(ABB72792.1), HvCBF1(AAL84170.1), ZmDBP4(ACO72995.1), HvCBF2(AAM13419.1), OsDREB1B(AAN02488.1), HvCBF3(ACC63520.1), ZmDREB1A(AAN76804.1), OsDREB1A(AEW67332.1), LpCBF3(AAX57275.1). **b** Alignment of the deduced amino acid sequences of A-6 subgroup of CmDREB6 and CiDREB6(AHJ08575.1), ZmDBF(AAM80486.1), RAP2–4(NP_1777931.1). The red line represents the conserved DNA-binding domain (AP2/ERF domain), the blue line represents two motifs of M1 and M2, 3 green box, 1 orange box and ▲ respectively represent 3 β-sheets, 1 α-helix and V14, L19

We cloned a 1,028 bp *CmDREB6* fragment from chrysanthemum. The largest open reading frame was 936 bp encoding a polypeptide of 311 amino acid residues (Additional file 1). The molecular weight of the putative protein was about 34.36 KDa, and the theoretical isoelectric point was 6.72. Phylogenic analysis of *CmDREB6* and DREB members from other species showed that *CmDREB6* is classified into the DREB A-6 subgroup, and is most closely related to *CiDREB6* from *Cichorium intybus* (Fig. 1a), displaying 73% similarity to *CiDREB6*. The amino acid alignment showed that CmDREB6 contains one typical AP2/ERF domain which includes 1 α-helices and 3 β-sheet with the 14th valine and 19th leucine conserved in the DREB A-6 subgroup (Fig. 1b).

### Subcellular localization of CmDREB6

**Fig. 2:**
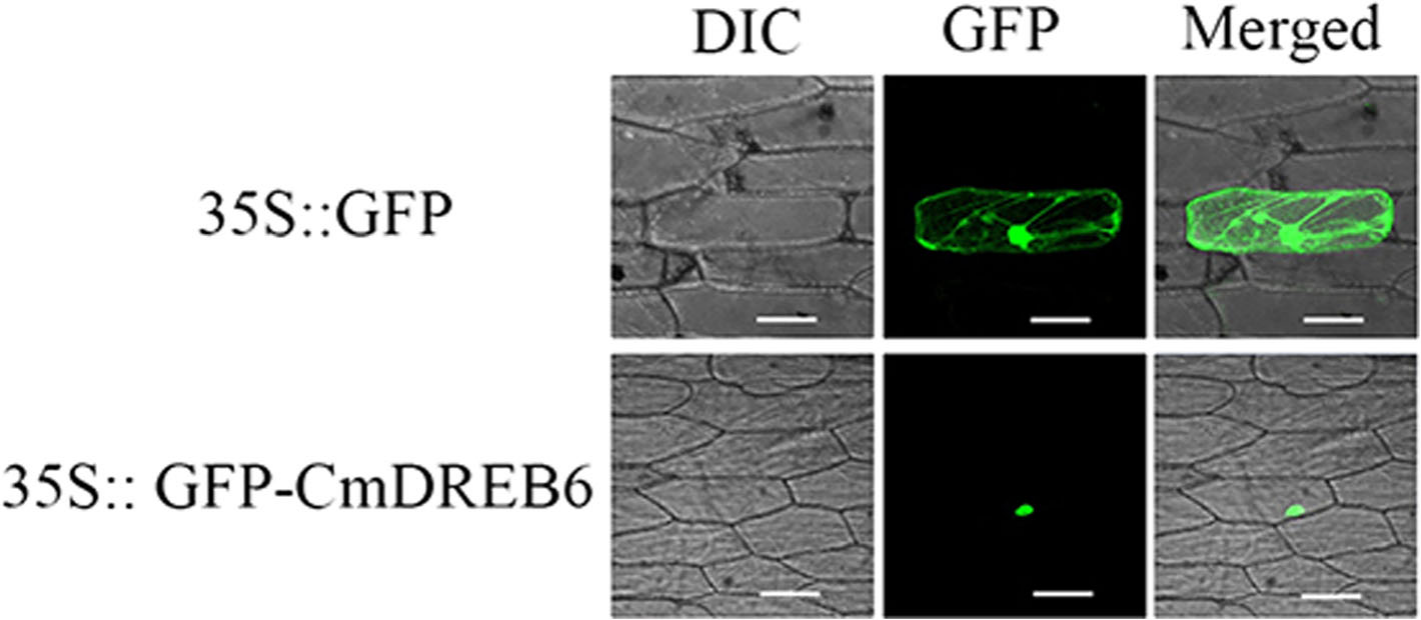
Subcellular localization of *CmDREB6.* Subcellular localization of transiently expressed *CmDREB6* products in onion epidermal cells. The upper row shows the control *35S::GFP* signal, and the lower row shows the signal of the *35S::GFP-CmDREB6* transgenes. The left panel shows bright field images, the middle one green fluorescence signals detected at 488 nm and the right one the merged Green Fluorescent Protein (GFP) and bright field images. Bar: 50 μm

The pMDC43-*GFP-CmDREB6* construct and empty vector pMDC43-GFP was transformed into onion epidermal cells via particle bombardment. GFP fluorescence of the control vector was evenly distributed throughout the observed onion epidermal cells (Fig. 2). In contrast, GFP fluorescence in the pMDC43-GFP-*CmDREB6* fusion protein was observed only in the nucleus of the onion epidermal cells (Fig. 2), indicating that CmDREB6 localized to the nucleus in vivo.

### Transactivation ability of CmDREB6 and its transcriptional activation domain analysis

**Fig. 3:**
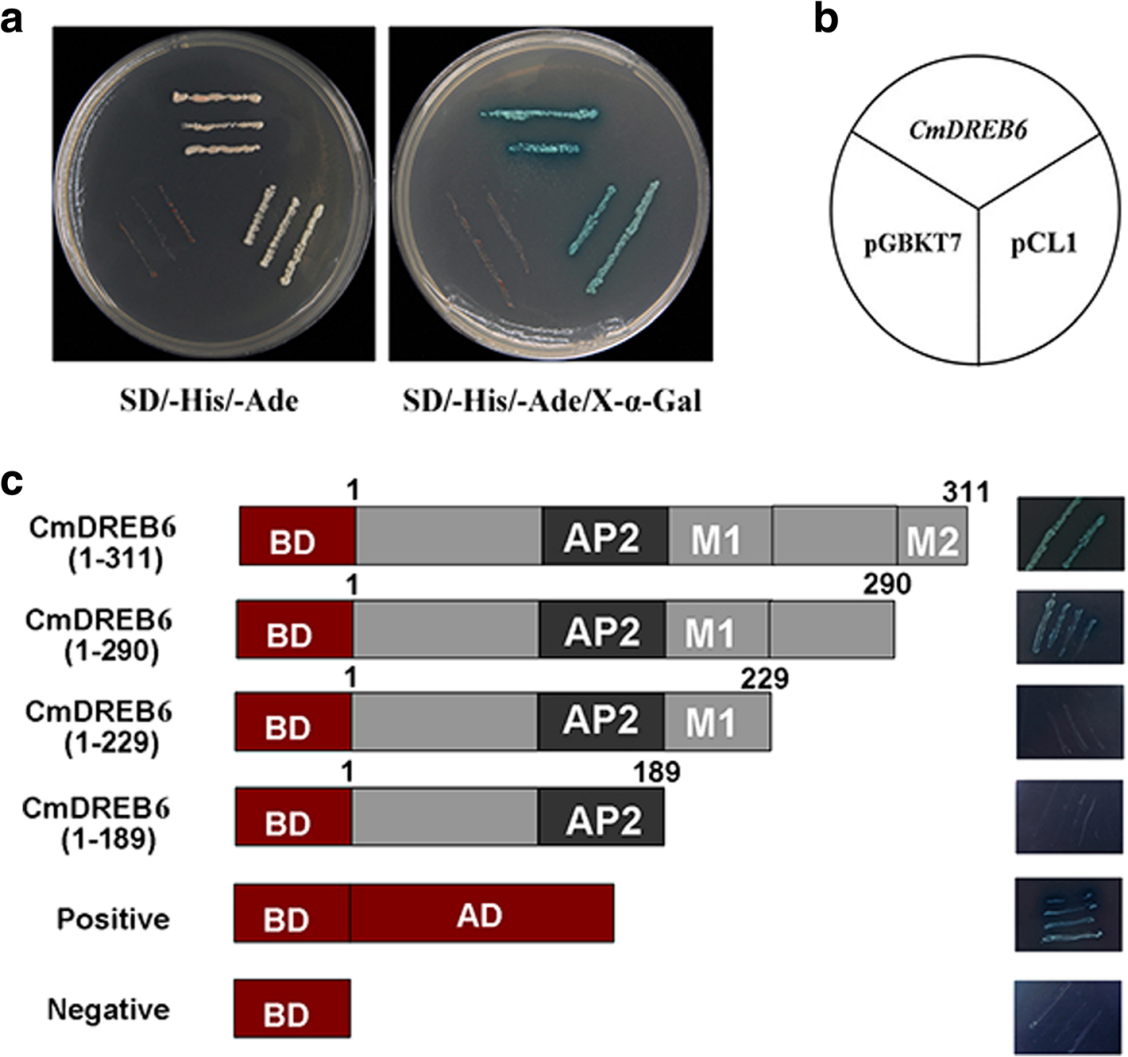
Transcriptional activity assay of *CmDREB6* protein and the analysis of transactivation activity domain of *CmDREB6*, (**a**): pGBKT7-CmDREB6; (**b**): distributed model; (**c**): transactivation activity domain analysis

The pGBKT7-*CmDREB6* and the control plasmids were introduced into the yeast strain Y2H gold. The results showed that the yeast strain containing the recombinant plasmid pGBKT7-*CmDREB6* was able to grow on SD / -Trp single-deficient medium, indicating that the recombinant plasmid was successfully transferred into the yeast strain Y2H gold; then the grown yeast was transferred to double-deficient medium SD / -His-Ade. The recombinant plasmid pGBKT7-*CmDREB6* grew normally, while the negative control strain containing the pGBKT7 plasmid did not grow on SD / -His-Ade, with the positive control pCL1 growing normally (Fig. 3a). The pGBKT7-*CmDREB6* colony on the double-defective plate supplemented with X-α-Gal turned blue (Fig. 3a), suggesting that pGBKT7-*CmDREB6* possessed transcriptional activation abilities.

To analyze the transcriptional activation domain of *CmDREB6*, the recombinant plasmid pGBKT7-*CmDREB6* (1–311 amino acid, full length), the truncated fragment constructs of pGBKT7-*CmDREB6* (1–290), pGBKT7-*CmDREB6* (1–229), pGBKT7-*CmDREB6* (1–189), and the pGBKT7 empty vector were subjected to yeast one hybrid assays. It was found that the yeast strains containing recombinant plasmids pGBKT7-*CmDREB6* (1–229) and pGBKT7-*CmDREB6* (1–189) could not grow normally, but pGBKT7-*CmDREB6* (1–311) and pGBKT7-*CmDREB6* (1–290) grew normally. The negative control strain containing the pGBKT7 empty plasmid did not grow on the SD / -His-Ade plate, but the positive control pCL1 grew normally (Fig. 3b). The results showed that the transcriptional activation domain of *CmDREB6* was located in the 230–290 amino acid at the C terminal.

### Tissue specific expression profiles and expression patterns of *CmDREB6* in response to heat stress

**Fig. 4:**
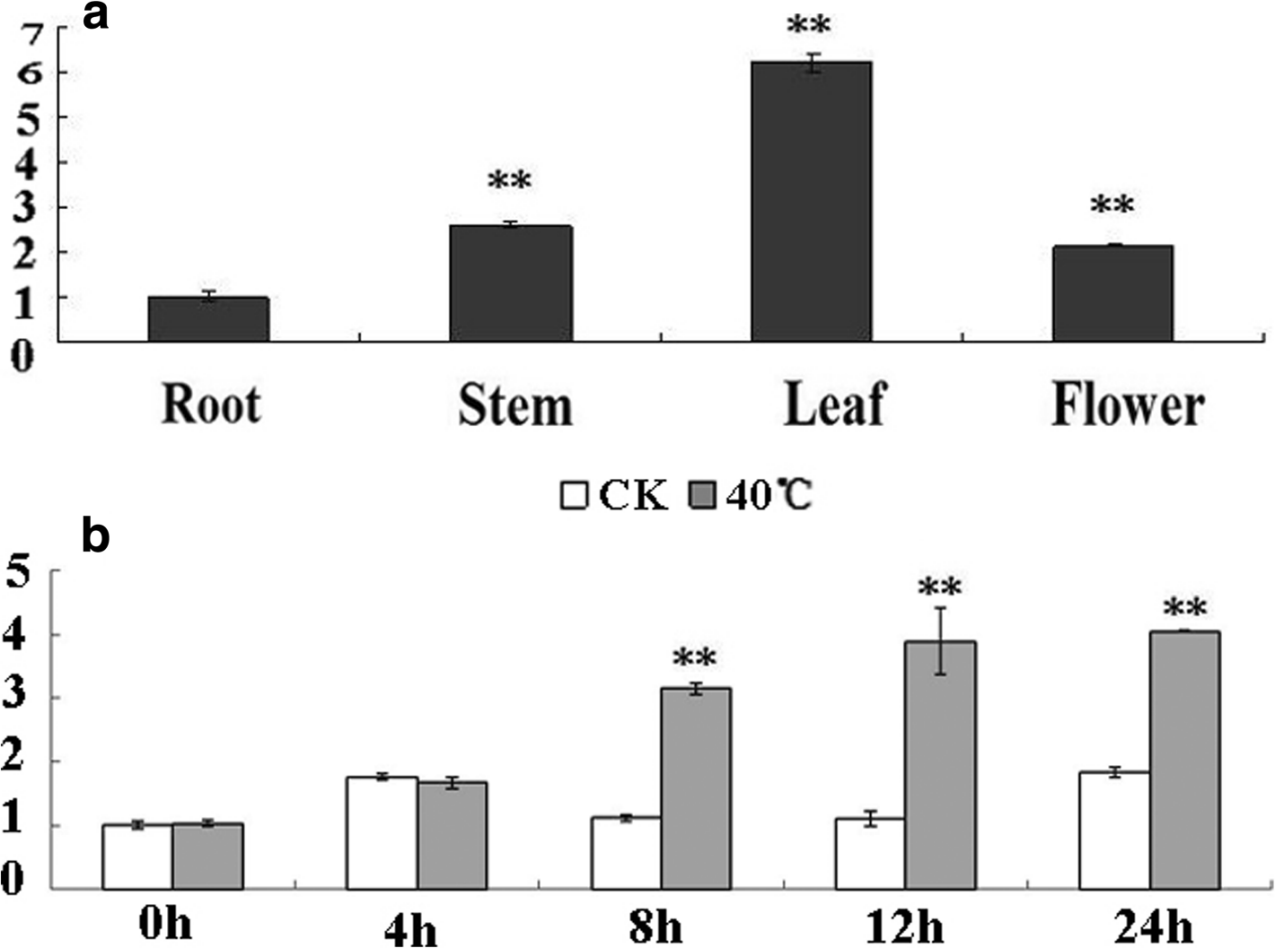
Expression patterns of *CmDREB6* in different organs and after 40 °C treatment. **a** Relative expression leveal of *CmDREB6* in root, stem, leaf and flower of chrysanthemum. The expression of *CmDREB6* was determined through qRT-PCR analysis. The expression level of *CmDREB6* in root was set to 1, the error bars indicate the SE from three replicate samples. Asterisks indicate significant differences in expression levels of *CmDREB6* in stem, leaf and flower compared with that in root. * represents significance at *p* < 0.05, ** represents significance at *p* < 0.01. **b** Relative expression leveal of *CmDREB6* after 40 °C treatment. The expression of *CmDREB6* was determined through qRT-PCR analysis. The expression level of *CmDREB6* in WT was set to 1, the error bars indicate the SE from three replicate samples. Asterisks indicate significant differences in expression level of *CmDREB6* under heat stress compared with WT at 0 h, 1 h, 3 h and 24 h, respectively, * represents significance at *p* < 0.05, ** represents significance at *p* < 0.01

RNA from root, stem, leaf and flower of chrysanthemum was extracted for analysis of the relative expression levels of *CmDREB6* in different tissues. The expression levels of *CmDREB6* was highest in leaves, followed by those in stem, flower and root in turn. The expression levels of *CmDREB6* in stem, leaves and flower was 1.6, 5.2 and 1.1 folds higher respectively than in the root (Fig. 4a). The expression levels of *CmDREB6* at 8 h, 12 h and 24 h after heat stress were 1.8, 2.6 and 1.2 folds higher than those at 0 h (before heat stress), respectively (Fig. 4b).

### *CmDREB6* overexpression enhanced the tolerance of chrysanthemum to heat stress

**Fig. 5:**
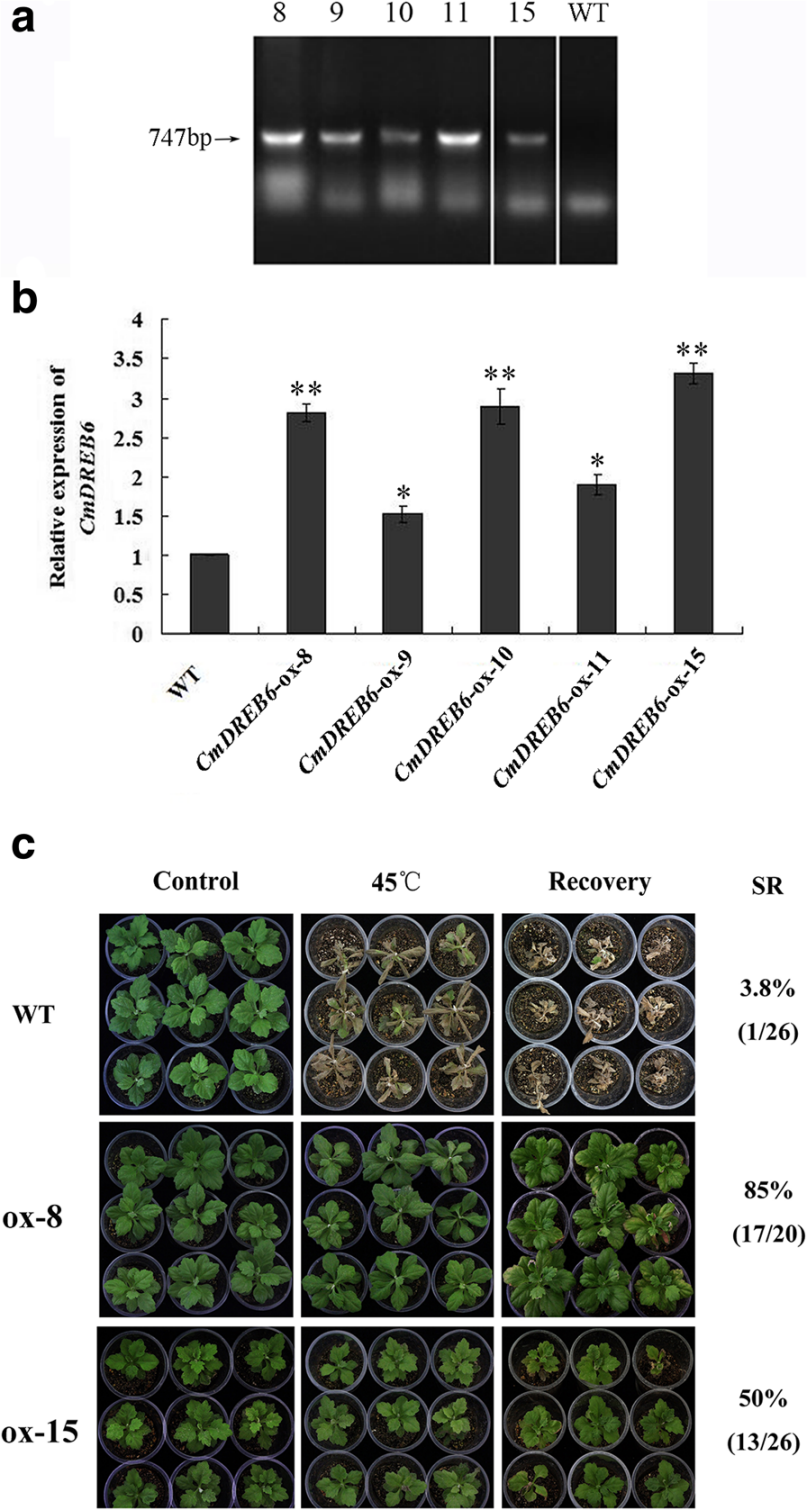
Analysis of phenotype and survival rate of two transgenic chrysanthemum and WT chrysanthemum. **a** PCR identification results of *CmDREB6* in the putative transgenic ‘Jinba’ by *Hyg* primers; M, 2000 Maker; 8, 9, 10, 11, 15 represents putative transgenic lines; 21 represents WT. **b** Relative expression level of *CmDREB6* in five putative transgenic lines; The expression of *CmDREB6* was determined through qRT-PCR analysis. The expression level of *CmDREB6* in WT was set to 1, the error bars indicate the SE from three replicate samples. Asterisks indicate significant differences in *CmDREB6* expression levels in putative transgenic lines compared with that in WT. * represents significance at *p* < 0.05, ** represents significance at *p* < 0.01. **c** The phenotype and survival rate of two transgenic chrysanthemum and WT plants under heat stress

Putative transgenic plants were verified by PCR amplification of hygromycin resistant gene. The expected bands with a fragment size of Ca. 750 bp were present in five putative transgenic lines, but not in WT plants (Fig. 5a; Additional file 2). The expression levels of *CmDREB6* plants in overexpressing lines were higher than those of WT ‘Jinba’; two overexpressing lines with higher expression levels of *CmDREB6*, ox-8 and ox-15, were selected for further heat stress tolerance assays (Fig. 5b). The results showed that WT plants wilted severely, and all the leaves of WT plants became wilted, shrunk and drooped, and especially the top leaves of WT were severely burned after 24 h heat-shock. In contrast, ox-8 and ox-15 lines displayed minor wilting, and most leaves remained green (Fig. 5c). After one-week recovery growth, almost all WT plants were scorched, and only one out of 26 plants survived with very weak growth, and the survival rate of WT plants was 3.8%, whereas the survival rate of ox-8 and ox-15 was 85% and 50%, respectively (Fig. 5c), indicating that *CmDREB6* conferred heat stress tolerance to chrysanthemum.

### *CmDREB6* over-expression induced the expression of *CmHsfA4*, *CmHSP90* and antioxidant enzymes encoding genes *CmSOD* and *CmCAT*

**Fig. 6:**
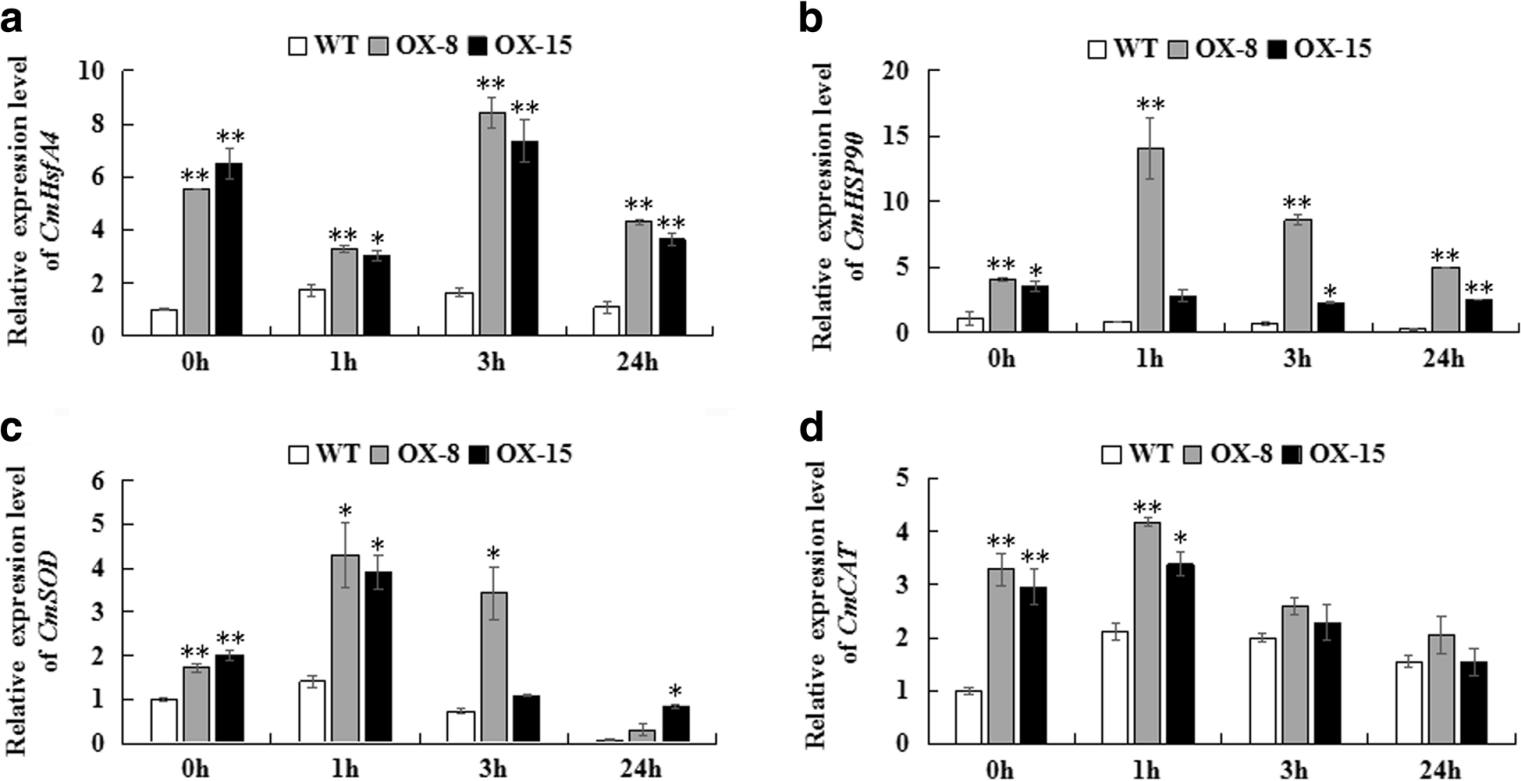
Relative expression levels of *CmHsfA4, CmHSP90, CmSOD* and *CmCAT.* **a** Relative expression levels of *CmHsfA4*; **b** Relative expression levels of *CmHsp90*; **c** Relative expression levels of *CmSOD*; **d** Relative expression levels of *CmCAT*. The expression of these genes was determined via qRT-PCR analysis. The expression level of *CmDREB6* in WT at 0 h was set to 1, the error bars indicate the SE from three replicate samples. Asterisks indicate significant differences in gene expression levels in *CmDREB6* overexpression lines compared with WT. * represents significance at *p* < 0.05, ** represents significance at *p* < 0.01

To dissect the pathways that lead to improved heat stress tolerance in chrysanthemum by overexpressing *CmDREB6*, the expression levels of *CmHsfA4*, *CmHSP90*, *CmSOD* and *CmCAT* were quantified in chrysanthemum under heat stress. Expression of *CmHsfA4* was enhanced by heat shock treatment in both WT and ox lines plants, the expression levels of *CmHsfA4* in ox-8, ox-15 plants were always higher than those in WT (Fig. 6a). The expression of *CmHSP90* increased in response to heat stress, the expression level of *CmHSP90* in both ox-8 and ox-15 plants remained higher than that of WT during heat stress (Fig. 6b). *CmDREB6* overexpression promoted expression of the reactive oxygen scavenging genes *CmSOD* and *CmCAT* in ox-8 and ox-15 plants; the expression levels of these two genes were higher than those in WT plants under heat stress (Fig. 6c, d).

## Discussion

### Chrysanthemum *CmDREB6* over-expression conferred heat tolerance

Members of the A-6 DREB family have been isolated from several species, and their roles in stress responses and development have been characterized. *CiDREB6* was mainly induced under intense heat and drought, but not by low temperature and high salt [9]. One of the eight members of the Arabidopsis DREB A-6 subgroup, *ERF055*, was expressed in the roots, stems, leaves, flowers and pods of Arabidopsis, with the highest expression levels in the pods; it has been verified that *ERF055* gene in Arabidopsis is involved in the development of embryo and postembryonic development [10]. The AP2/ERF transcription factor WIND1 (WOUND INDUCED DEDIFFERENTIATION 1), which belongs to the A-6 DREB subgroup, promotes cell dedifferentiation in Arabidopsis, and induces callus formation in rapeseed, tomato, and tobacco [11]. Other members of the A-6 subgroup were successively isolated in different species. For example, in *Jatropha curcas* L., expression of *JcDREB* was induced by cold, salt and drought stress, but not by ABA (Abscisic acid), and overexpression of *JcDREB* in Arabidopsis enhances salt tolerance and freezing tolerance [12]. *GhDBP2* was expressed at high levels in the leaves of *Gossypium hirsutum*, and was strongly induced by drought, high salt, low temperature and ABA [13]. In *Nicotiana tabacum*, overexpressing the *Suaeda salsa SsDREB*, a member of A-6 subgroup, tolerance to salt and drought increased, as well as the growth rate, chlorophyll content and photosynthetic rate, protein level and soluble sugar content compared to WT plants [14]. The expression of *MsDREB6.2* from *Malus sieversii* Roem is strongly induced by drought and salinity stress, and is expressed at high levels in roots. Overexpression of *MsDREB6.2* resulted in cytokinin-deficient developmental phenotypes by enhancing *MdCKX4*a expression and enhanced drought tolerance in transgenic apple plants [15]. These data indicate that *DREB* transcription factors could be used to improve the tolerance of plants to abiotic stress, and different DREB transcription factors have different members and species specificity [16]. In this study, the expression of the DREB A-6 subgroup member *CmDREB6* was highest in leaves, and was induced by high temperatures, suggesting that *CmDREB6* may play a role in response to heat stress. In agreement with heat inducible expression, *CmDREB6-*overexpressed chrysanthemum enhanced the tolerance to heat (Fig. 5), suggesting that *CmDREB6* confers heat tolerance.

### *CmDREB6* enhanced heat tolerance of chrysanthemum by upregulating the expression of *CmHsfA4* and *CmHSP90*

*HSFs* (Heat shock factors) play important roles in the response to heat stress [17]. *HsfA3* acts as a thermo-stable regulator under the control of the *DREB2A* pathway, is downstream of the *DREB2A* stress-modulating mechanism, and regulates the expression of many heat-inducible genes [18]. More evidence showed that DREB2A activates the *HsfA3* and subsequently regulates the expression of *HSP* genes involved in the early phases of the HSR, and DREB2C transactivates DRE-dependent transcription of *HsfA3* inducing the expression of the downstream gene *HSPs*, thereby enhancing thermotolerance in the late phases of HSR [19, 20]. Recent studies have shown that *HsfA2* and *HsfA3* function in the same heat regulation pathway, and *HsfA2* plays a dominant role over *HsfA3* [21]. HsfA4 is a potent activator of heat stress gene expression [22]. However, the regulation of *HsfA4* by DREB has not been reported previously. In the present study, induction of *CmHsfA4* was observed in *CmDREB6* overexpressed plants either in non-stressed or heat stressed plants (Fig. 6), suggesting the DREB A-6 subfamily member *CmDREB6*, might enhance heat tolerance in chrysanthemum by activating the expression of *CmHsfA4*. However, whether CmDREB6 binds to the promoter of *CmHsfA4* directly remains to be determined.

HSPs are important molecular chaperones, widely mediating stress signals [23]. In the absence of stress, HSP and HSF exist together as molecular chaperones. After a heat shock reaction, plants produce large amounts of toxic proteins, and HSPs are released from HSFs and then bind to toxin proteins [24]. Many HSP proteins are known to act as molecular chaperones for the protection of thermo-labile proteins against heat-induced denaturation in plant cells [25–27]. In this study, expression of *CmHSP90* was induced in *CmDREB6* overexpressing plants, which might protect chrysanthemum from heat induced damage. Members of the HsfA subclass A4 have been shown to serve as transcriptional activators for *HSP* genes [19, 28, 29]. Whether induction of *CmHsfA4* in *CmDREB6* overexpressing plants upregulates the expression of *CmHSP70* and *CmHSP90* remains to be elucidated. The *MsHSFA4* gene in *Medicago sativa* was not expressed under non-stress conditions but its expression was induced by heat shock, similarly to *AtHSFA4a* expression [30].

### *CmDREB6* enhanced heat tolerance of chrysanthemum by regulating ROS homogenesis

Heat stress may induce the generation and reactions of activated oxygen species (ROS) including singlet oxygen (^1^O_2_), superoxide radical (O_2_^−^), hydrogen peroxide (H_2_O_2_) and hydroxyl radical (OH^−^) [31]. ROS causes the autocatalytic peroxidation of membrane lipids and pigments, thus leading to the loss of membrane semi-permeability and modifying its functions [1]. In response, plants will then produce antioxidant enzymes, such as superoxide dismutase (SOD), catalase (CAT) to mitigate the damage to the cell membrane and improve heat resistance [1]. *HSP90* was found to be involved in the regulation of *HsfA2* expression in response to oxidative stress, where the heat shock response promotes expression of *SOD*, while the active oxygen scavenging gene *CAT* expression was not significantly altered [24]. Overexpression of *HsfA4A* could prevent oxidative damage in Arabidopsis and enhance tolerance not only to salt but also to osmotic stress, paraquat, H_2_O_2_ and anoxia [32]. The *ThDREB* from *Tamarix hispida* could effectively improve tolerance to salt and drought stress by enhancing the antioxidase activity that keeps ROS accumulation at low levels, thus facilitating scavenging [33]. Ectopic expression of *EgDREB1* from *Elaeis guineensis* enhanced expression of tomato peroxidase (*LePOD*), ascorbate peroxidase (*LeAPX*), catalase (*LeCAT*), superoxide dismutase (*LeSOD*), heat shock protein 70 (*LeHSP70*) in tomato seedlings under PEG treatment and cold stress [34]. The *AtDREB1B* transgenic plants generally displayed lower levels of malondialdehyde (MDA) but higher levels of superoxide dismutase (SOD), catalase (CAT), and peroxidase (POD) activities than the WT under drought stress [35]. Similarly, *CmDREB6* overexpression could promote the expression of ROS pathway related genes *CmSOD* and *CmCAT* in chrysanthemum, suggesting that *CmDREB6* enhances heat tolerance of chrysanthemum by regulating ROS homeostasis.

In addition, over accumulation of stress-related transcription regulators could be detrimental to growth and is not deemed preferable [36, 37]. In this study, growth retardation was observed in the *CmDREB6* ox-15 line with highest expression levels of *CmDREB6*. Compared to the WT plants, the tolerance of ox-15 line to heat stress and the activation of the expression of downstream genes are not as obvious as those of the ox-8 line, suggesting that over accumulation of *CmDREB6* retarded growth might affect tolerance to heat stress.

## Conclusions

The expression of the DREB A-6 subgroup member *CmDREB6* was induced by high temperatures. And *CmDREB6*-overexpressed chrysanthemum enhanced the tolerance to heat, suggesting that *CmDREB6* confers heat tolerance. Induction of C*mHsfA4* was observed in *CmDREB6* overexpressed, and *CmHSP90* was induced in *CmDREB6* overexpressing plants, which might protect chrysanthemum from heat induced damage. *CmDREB6* overexpression could promote the expression of ROS pathway related gene *CmSOD* and *CmCAT* in chrysanthemum, suggesting that *CmDREB6* enhances heat tolerance of chrysanthemum by regulating ROS homeostasis.

## Methods

### Plant materials and growth conditions

Cuttings of the chrysanthemum variety ‘Jinba’ were obtained from the Chrysanthemum Germplasm Resource Preserving Center (Nanjing Agricultural University, Nanjing, China). The cuttings were potted into a 1:2 (*v*/v) mixture of garden soil and vermiculite, and were maintained in a greenhouse with a relative humidity of 80%, light intensity 100 μmol·m^− 2^·s^− 1^ and a 16 h/8 h (light/dark) photoperiod, with day and night temperatures of 23 °C and 18 °C, respectively.

### Isolation and sequence analysis of *CmDREB6*

Total RNA was isolated from chrysanthemum leaves using the RNAiso reagent (Takara, Tokyo Japan) and following the manufacturer’s protocol. A 1 μg aliquot of the resulting RNA treated with RNase-free DNase I was included as the template for 1st strand cDNA synthesis, using Super Script III reverse transcriptase (Invitrogen, Carlsbad, CA, USA). The *CmDREB6* open reading frame (ORF) was amplified using *CmDREB6*-F/R primers (Additional file 3). The PCR product purified using a Biospin Gel Extraction kit (Bio Flux, Hangzhou, China) was introduced into pMD19-T (Takara) for sequencing. The *CmDREB6* sequence was aligned with its homologs using the DNAMANV6 software [38], and a phylogenic tree was generated using MEGA5.0 software based on the neighbor-joining method and 1,000 bootstrap replicates. The polypeptides sequences of other DREB members from other species were obtained from the NCBI website (https://www.ncbi.nlm.nih.gov).

### Tissues specific expression patterns of *CmDREB6* and its expression profiles in response to heat stress

Roots, stems and leaves were harvested from four-week old chrysanthemum plants to characterize the tissue specific expression profile of *CmDREB6* transcription. The heat stress experiment (40 °C) was conducted as previously described [39]. The second leaf (counted from the shoot apex) at 0 h, 4 h, 8 h, 12 h and 24 h after heat stress, respectively, was sampled for RNA isolation. 1st strand cDNA was transcribed as previously described. Transcript abundance was detected by quantitative real time PCR (qPCR) using SYBR® Premix Ex Taq™ II (Tli RNaseH Plus) (Takara) and the primer pair *CmDREB6-RT-F/R* (Additional file 3). The primer pair *CmEF1α-F/R* (Additional file 3) was used to amplify the reference gene *CmEF1α*. Fold changes were calculated using the 2^−ΔΔCt^ method [40]. Each experiment included three biological replicates.

### Sub-cellular localization and transcriptional activation assay of CmDREB6

The *CmDREB6* ORF was amplified using a Phusion® High Fidelity PCR Kit (New England Biolabs, Ipswich, MA, USA) with the primer pair CmDREB6-Nde-F/-BamH-R (Additional file 3). The resulting amplification was digested by *Nde*I/*BamH*I, and then ligated into pENTR™1A (Invitrogen) to form the construct pENTR™1A*-CmDREB6*.

For the subcellular assay, *CmDREB6* was further inserted into the destination vector pMDC43 or pDEST-GBKT7 via LR reaction. The pMDC43*-GFP*-*CmDREB6* construct and the empty pMDC43 vector were introduced into onion epidermal cells via particle bombardment (PDS-1000; Bio-Rad). Transformed cells were held for 16 h on Murashige and Skoog (MS) medium in the dark, and then GFP (Green fluorescent protein) florescence was observed under a confocal laser scanning microscope.

For the transcriptional activation assay, *CmDREB6* was inserted into the destination vector pDEST-GBKT7 via LR reaction. The pDEST-GBKT7 vector with different segments of *CmDREB6* (1–311, 1–189, 1–229, 1–290 amino acid) was constructed to screen the transactivation region of the protein. Clones were sequenced to verify the inserts were correct. The recombinant plasmids pGBKT7-*CmDREB6* or pGBKT7 empty vector (negative control) or pCL1 (positive control) were transformed into a Y2H gold yeast strain, then plated on SD / -Trp plates and cultured at 30 °C for 3 days. Yeast colonies were then transferred to medium SD / -His-Ade and cultured in the dark at 30 °C for 3 days. The growth of yeast was observed and photographed.

### Regeneration of *CmDREB6* overexpressing chrysanthemum plants

To identify the function of *CmDREB6*, the vector of pMDC43*-GFP*-*CmDREB6* driven by 35S promoter was introduced into chrysanthemum ‘Jinba’ by Agrobacterium-mediated leaf disc infection [41]. Putative transgenic plants were verified by PCR analysis using *Hyg* (*hygromycin*) *F/R* primers, and over-expression of *CmDREB6* in transgenic plants was detected via qPCR using primers *CmDREB6-RT-F/R*.

### Heat stress tolerance assay for *CmDREB6* overexpressing chrysanthemum plants

Over-expression lines and WT plants were planted in the same batch (soil: vermiculite; 1: 1, *v*/v). For the heat tolerance assay, chrysanthemum seedlings at the 6–8 leaves stage were exposed to 45 °C for 24 h; heat stressed plants were then transferred to 22 °C and left to recover for one-week [4]. Phenotypic changes before and after heat treatment respectively were documented, and survival rates were calculated.

### Gene expression profiles in *CmDREB6* overexpressing chrysanthemum plants

To further dissect the mechanisms involved in *CmDREB6* regulated heat tolerance in chrysanthemum, the third leaves (counted from the shoot apex) of heat stressed chrysanthemum and WT plants were sampled at 0 h, 1 h, 3 h, 24 h after exposure to heat treatment. Total RNA isolation and cDNA were prepared as detailed above. Expression levels of the heat stress-related genes *CmHsfA4* (*CmHsfA4 F/R*), *CmHSP90* (*CmHSP90 F/R*) and antioxidant enzymes encoding genes of *CmSOD* (*CmSOD F/R*) and *CmCAT* (*CmCAT F/R*) were quantified. All the primers used are listed in Additional file 3.

### Statistical analysis

Results are expressed as mean ± standard error. Statistical significance was determined by SPSS 19.0 amongst the means of WT and transgenic plants, and a one-way analysis of variance using LSD (least significant diferrence) multiple range test was employed to identify treatment means that differed statistically.

## Additional files

Additional file 1: **Text S1.** The amino acid sequence of CmDREB6 under accession No. MG199593. (DOC 22 kb)

Additional file 2: **Figure S1.** The electrophoresis analysis of PCR products of hygromycin resistant gene *HptII* in the putative *CmDREB6* transgenic ‘Jinba’. (TIF 59 kb)

Additional file 3: **Table S1.** The sequence of primers used in this research. (DOC 24 kb)

## Abbreviations

ABA: Abscisic acid
AP2/EREBP: Ethylene-responsive element binding proteins
AP2/ERF: Ethylene-responsive factor
CAT: catalase
DREB: dehydration-responsive element-binding
GFP: Green fluorescent protein
HSE: heat shock elements
HSF: heat shock factor
HSP: heat shock protein
HSR: Heat shock response
*Hyg*: hygromycin
MDA: Malondialdehyde
mRNA: Messenger ribonucleic acid
MS: Murashige and Skoog
ORF: Open reading frame
PCR: Polymerase chain reaction
POD: Peroxidase
ROS: Reactions oxygen species
SOD: Superoxide dismutase
WIND1: WOUND INDUCED DEDIFFERENTIATION 1
WT: Wild-type

## References

[1] Xu S, Li J, Zhang X, Wei H, Cui L (2006). Effects of heat acclimation pretreatment on changes of membrane lipid peroxidation, antioxidant metabolites, and ultrastructure of chloroplasts in two cool-season turfgrass species under heat stress. Environ Exp Bot.

[2] Larkindale J, Hall JD, Knight MR, Vierling E (2005). Heat stress phenotypes of Arabidopsis mutants implicate multiple signaling pathways in the acquisition of thermotolerance. Plant Physiol.

[3] Liu Q, Kasuga M, Sakuma Y, Abe H, Miura S, Yamaguchi-Shinozaki K (1998). Two transcription factors, *DREB1* and *DREB2*, with an EREBP/AP2 DNA binding domain separate two cellular signal transduction pathways in drought- and low-temperature-responsive gene expression, respectively, in *Arabidopsis*. Plant Cel.

[4] Hong B, Ma C, Yang Y, Wang T, Yamaguchi-Shinozaki K, Gao J (2009). Over-expression of *AtDREB1A* in chrysanthemum enhances tolerance to heat stress. Plant Mol Biol.

[5] Wahid A, Gelani S, Ashraf M, Foolad MR (2007). Heat tolerance in plants: an overview. Environ Exp Bot.

[6] Pelham HR (1982). A regulatory upstream promoter element in the drosophila hsp 70 heat-shock gene. Cell.

[7] Hartl FU, Hayerhartl M (2002). Molecular chaperones in the cytosol: from nascent chain to folded protein. Science.

[8] Chen S, Cui X, Chen Y, Gu C, Miao H, Gao H (2011). *CgDREBa* transgenic chrysanthemum confers drought and salinity tolerance. Environ Exp Bot.

[9] Liang M, Chen D, Lin M, Zheng Q, Huang Z, Lin Z, Zhao G (2014). Isolation and characterization of two *DREB1* genes encoding dehydration-responsive element binding proteins in chicory (*Cichorium intybus*). Plant Growth Regul.

[10] Teng F. Molecular dissection of Arabidopsis AP2/ERF family gene ERF055 in regulation of the development of the shoot apical meristem [D]. Tai'an: Shandong agricultural university; 2013. (In Chinese)

[11] Iwase A, Mitsuda N, Ikeuchi M, Ohnuma M, Koizuka C, Kawamoto K (2013). Arabidopsis *WIND1* induces callus formation in rapeseed, tomato, and tobacco. Plant Signal Behav.

[12] Tang M, Liu X, Deng H, Shen S (2011). Over-expression of *JcDREB*, a putative AP2/EREBP domain-containing transcription factor gene in woody biodiesel plant Jatropha curcas, enhances salt and freezing tolerance in transgenic *Arabidopsis thaliana*. Plant Sci.

[13] Huang B, Jin L, Liu JY (2008). Identification and characterization of the novel gene *GhDBP2* encoding a DRE-binding protein from cotton (*Gossypium hirsutum*). J Plant Physiol.

[14] Zhang X, Liu X, Wu L, Yu G, Wang X, Ma H. The SsDREB transcription factor from the succulent halophyte Suaeda salsa enhances abiotic stress tolerance in transgenic tobacco. Int J Genomics. 2015;2015(5):1–13. 10.1155/2015/875497 PMC4609462 26504772

[15] Xiong L, Xiao G, Qi W, Wang Y, Di Z, Yao L (2017). Overexpression of *MsDREB6.2* results in cytokinin-deficient developmental phenotypes and enhances drought tolerance in transgenic apple plants. Plant J.

[16] Zhou W, Jia CG, Wu X, Hu RX, Yu G, Zhang XH (2016). *ZmDBF3*, a novel transcription factor from maize ( *Zea mays* L.), is involved in multiple abiotic stress tolerance. Plant Mol Biol Rep.

[17] Li C, Chen Q, Gao X, Qi B, Chen N, Xu S, Wang X (2005). *AtHsfA2* modulates expression of stress responsive genes and enhances tolerance to heat and oxidative stress in *Arabidopsis*. Sci China Ser C Life Sci.

[18] Nishizawa A, Yabuta Y, Yoshida E, Maruta T, Yoshimura K, Shigeoka S (2006). Arabidopsis heat shock transcription factor A2 as a key regulator in response to several types of environmental stress. Plant J.

[19] Schramm F, Ganguli A, Kiehlmann E, Englich G, Walch D, Koskull-Döring P (2006). The heat stress transcription factor *HsfA2* serves as a regulatory amplifier of a subset of genes in the heat stress response in *Arabidopsis*. Plant Mol Biol.

[20] Chen H, Hwang JE, Lim CJ, Kim DY, Lee SY, Lim CO (2010). Arabidopsis DREB2C functions as a transcriptional activator of *HsfA3* during the heat stress response. Biochem Bioph Res Co.

[21] Li XD, Wang XL, Cai YM, Wu JH, Mo BT, Yu ER (2017). Arabidopsis heat stress transcription factors A2 *( HSFA2 )* and *A3 ( HSFA3 )* function in the same heat regulation pathway. Acta Physiol Plant.

[22] Baniwal SK, Chan KY, Scharf KD, Nover L (2007). Role of heat stress transcription factor *HsfA5* as specific repressor of HsfA4. J Biol Chem.

[23] Hahn A, Bublak D, Schleiff E, Scharf KD (2011). Crosstalk between Hsp90 and Hsp70 chaperones and heat stress transcription factors in tomato. Plant Cell.

[24] Nishizawayokoi A, Tainaka H, Yoshida E, Tamoi M, Yabuta Y, Shigeoka S (2010). The 26S proteasome function and Hsp90 activity involved in the regulation of *HsfA2* expression in response to oxidative stress. Plant Cel Physiol.

[25] Wang W, Vinocur B, Shoseyov O, Altman A (2004). Role of plant heat-shock proteins and molecular chaperones in the abiotic stress response. Trends Plant Sci S.

[26] Basha E, Jones C, Wysocki V, Vierling E (2010). Mechanistic differences between two conserved classes of small heat shock proteins found in the plant cytosol. J Biol Chem.

[27] Waters ER (2013). The evolution, function, structure, and expression of the plant sHSPs. J Exp Bot.

[28] Pérezsalamó I, Papdi C, Rigó G, Zsigmond L, Vilela B, Lumbreras V (2014). The heat shock factor A4A confers salt tolerance and is regulated by oxidative stress and the mitogen-activated protein kinases MPK3 and MPK6. Plant Physiol.

[29] Xue GP, Sadat S, Drenth J, Mcintyre CL (2014). The heat shock factor family from Triticum aestivum in response to heat and other major abiotic stresses and their role in regulation of heat shock protein genes. J Exp Bot.

[30] Friedberg JN, Bowley SR, Mckersie BD, Gurley WB, Czarnecka-Verner E (2006). Isolation and characterization of class A4 heat shock transcription factor from alfalfa. Plant Sci.

[31] Liu X, Huang B (2000). Heat stress injury in relation to membrane lipid peroxidation in creeping Bentgrass. Crop Sci.

[32] Pérez Salamó I (2014). Functional characterization of the Arabidopsis heat shock factor A4A, identified by a novel genetic screen. Geochim Cosmochim Acta.

[33] Yang G, Yu L, Zhang K, Zhao Y, Guo Y, Gao C (2017). A *ThDREB* gene from Tamarix hispida improved the salt and drought tolerance of transgenic tobacco and T. Hispida. Plant Physiol Bioch..

[34] Azzeme AM, Abdullah SN, Aziz MA, Wahab PE (2016). Oil palm drought inducible *DREB1* induced expression of DRE/CRT- and non-DRE/CRT-containing genes in lowland transgenic tomato under cold and PEG treatments. Plant Physiol Bioch.

[35] Wei T, Deng K, Gao Y, Liu Y, Yang M, Zhang L (2016). Arabidopsis *DREB1B* in transgenic salvia miltiorrhiza increased tolerance to drought stress without stunting growth. Plant Physiol Bioch..

[36] Ogawa D, Yamaguchi K, Nishiuchi T (2007). High-level overexpression of the Arabidopsis *HsfA2* gene confers not only increased themotolerance but also salt/osmotic stress tolerance and enhanced callus growth. J Exp Bot.

[37] Yan Z, Zhi W, Jing Y, Wang L, Xia L, Liu Y (2009). Ectopic over-expression of *BhHsf1*, a heat shock factor from the resurrection plant Boea hygrometrica, leads to increased thermotolerance and retarded growth in transgenic Arabidopsis and tobacco. Plant Mol Biol.

[38] MA L GB, NP B RC, PA M HM (2007). Clustal W and Clustal X version 2.0. Bioinformatics.

[39] Song A, Li P, Jiang J, Chen S, Li H, Zeng J (2014). Phylogenetic and transcription analysis of chrysanthemum *WRKY* transcription factors. Int J Mol Sci.

[40] Kenneth J, Livak TD (2001). Analysis of relative gene expression data using real-time quantitative PCR and the 2^−ΔΔCt^ method. Method.

[41] Jong JD, Rademaker W, Wordragen MFV (1993). Restoring adventitious shoot formation on chrysanthemum leaf explants following cocultivation with agrobacterium tumefaciens. Plant Cell Tiss Org.

