# Supplementary material for: Over-expression of chrysanthemum CmDREB6 enhanced tolerance of chrysanthemum to heat stress
Source: BMC Plant Biol. 2018 Sep 4;18:178. doi: 10.1186/s12870-018-1400-8 (PMC6122619; doi:10.1186/s12870-018-1400-8)
Supplement: Supplementary file 1 — Text S1. The amino acid sequence of CmDREB6 under accession No. MG199593. (DOC 22 kb) [file 12870_2018_1400_MOESM1_ESM.doc]

**Amino acid sequence of CmDREB6 under accession of MG199593**

MASATMDLWNLDFQQFNGGELMDALEPFIKQDQNYQNTLPYSSPSTSYPSPTQLQSGFYPDPSFTHDPFFNLQPVSSFGLNQLTPSQIHQIQAQINLPNFSPTYLGPKPIPMKQTGSGSPTKPTKLYRGVRQRHWGKWVAEIRLPKNRTRLWLGTFDTAEEAALAYDIAAYKLRGDYARLNFPHLRHNGSHIAGACGDYAPLHSSVDAKLQAICQNLAEGKSVDGGKKKSQRRSTVVKPPVKHPATVVEQPEVVKVEGSSESSSSGGSSPVSEISELSFPEFTAEDGGAWGASDCFLLEKYPSYEIDWGSI
