# Supplementary figures and images for: Over-expression of chrysanthemum CmDREB6 enhanced tolerance of chrysanthemum to heat stress
Source: BMC Plant Biol. 2018 Sep 4;18:178. doi: 10.1186/s12870-018-1400-8 (PMC6122619; doi:10.1186/s12870-018-1400-8)

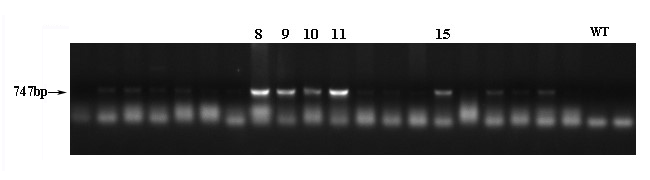

Supplement: Supplementary file 2 — Figure S1. The electrophoresis analysis of PCR products of hygromycin resistant gene HptII in the putative CmDREB6 transgenic ‘Jinba’. (TIF 59 kb) [file 12870_2018_1400_MOESM2_ESM.tif]
