# Supplementary material for: Over-expression of chrysanthemum CmDREB6 enhanced tolerance of chrysanthemum to heat stress
Source: BMC Plant Biol. 2018 Sep 4;18:178. doi: 10.1186/s12870-018-1400-8 (PMC6122619; doi:10.1186/s12870-018-1400-8)
Supplement: Supplementary file 3 — Table S1. The sequence of primers used in this research. (DOC 24 kb) [file 12870_2018_1400_MOESM3_ESM.doc]

**Table.S1 The sequence of primers used in this research**

| **Primer** | **Sequence(5’-3’)** |
| --- | --- |
| *CmDREB6-F* | ATGGCTTCAGCTACAATGGACTTAT |
| *CmDREB6-R* | ATATACCCTCATAAACACTGCCACG |
| *EF1α-F* | TTTTGGTATCTGGTCCTGGAG |
| *EF1α-R* | CCATTCAAGCGACAGACTCA |
| *CmDREB6-Nde I-F* | CTGCATATGATGGCTTCAGCTACAATGGAC |
| *CmDREB6-BamH I-R* | CGCGGATCCCGATAGAACCCCAATCAATCT |
| *CmDREB6-BamH I-R(1-189)* | CGCGGATCCCCCCATTGTGGCGCAAGTGAGG |
| *CmDREB6-BamH I-R(1-229)* | CGCGGATCCCTTTCTTCTTACCACCGTCGAC |
| *CmDREB6-BamH I-R（1-290）* | CGCGGATCCC CCACGCGCCGACGTCTTCCGC |
| *CmDREB6-RT-F* | AACAATTCAATGGTGGTG |
| *CmDREB6-RT-R* | GTCGTGGGTGAAAGATGG |
| *Hyg-F* | CTTCTACACAGCCATCGGTCCAG |
| *Hyg-R* | CGGAAGTGCTTGACATTGGGGAG |
| *CmHsfA4-F* | ATGATGATGAGATTGGTACGGC |
| *CmHsfA4-R* | TCAAAAAGAACACATAATAACATACA |
| *CmHSP90-F* | GAACTTGGTGACACGGATGA |
| *CmHSP90-R* | ATGCAGCATTTAAGTCCTTG |
| *CmSOD-F* | GCATGTCAACTGGTCCTCATTACAA |
| *CmSOD-R* | TCACGAAAGTAAGCCTTGCTATCCC |
| *CmCAT-F* | TTGTTGGAGGACGAAG |
| *CmCAT-R* | CAGCGGCAAGATGTCC |
